# Making the most of existing antimalarial medicines: a single dose cure with sulfadoxine–pyrimethamine plus artesunate–pyronaridine

**DOI:** 10.1186/s12936-025-05559-4

**Published:** 2025-09-30

**Authors:** Ghyslain Mombo-Ngoma, Michael Ramharter, Rella Zoleko Manego, Bertrand Lell, Quique Bassat, Pedro Aide, Oumou Maiga Ascofare, Timothy N. C. Wells, Abdoulaye Djimde, Francine Ntoumi, Peter G. Kremsner

**Affiliations:** 1 Centre de Recherches Médicales de Lambaréné (CERMEL), BP 242, Lambaréné, Gabon; 2 Drug Implementation Research Group, Bernhard Nocht Institute for Tropical Medicine, & I. Dep. of Medicine, University Medical Centre Hamburg-Eppendorf, Bernhard-Nocht-Strasse 74, 20359 Hamburg, Germany; 3 German Centre for Infection Research (DZIF), Partner Site Hamburg-Lübeck-Borstel-Riems, Hamburg, Germany; 4 Center for Tropical Medicine, Bernhard-Nocht Institute for Tropical Medicine & I. Dept. of Medicine, University Medical Centre Hamburg-Eppendorf, Hamburg, Germany; 5 Department of Medicine I, Division of Infectious Diseases and Tropical Medicine, Medical University of Vienna, 1090 Vienna, Austria; 6 ISGlobal, Hospital Clínic-Universitat de Barcelona, Barcelona, Spain; 7 Centro de Investigação em Saúde de Manhiça (CISM), Maputo, Mozambique; 8 ICREA, Passeig de Lluís Companys 23, 08010 Barcelona, Spain; 9 Pediatrics Department, Hospital Sant Joan de Déu, Universitat de Barcelona, Esplugues, Barcelona Spain; 10 CIBER de Epidemiología y Salud Pública, Instituto de Salud Carlos III, Madrid, Spain; 11 Kumasi Center for Collaborative Research, Kumasi, Ghana; 12 Department of Infectious Diseases Epidemiology, Bernhard Nocht Institute for Tropical Medicine, Hamburg, Germany; 13 Medicines for Malaria Venture, Geneva, Switzerland; 14 Malaria Research and Training Centre (MRTC), Faculty of Pharmacy, Université Des Sciences, Des Techniques et des Technologies de Bamako (USTTB), Bamako, Mali; 15 Pathogens Genomic Diversity Network Africa, Imm. Gwancoura, Sotuba, Bamako, Mali; 16 Fondation Congolaise Pour La Recherche Médicale (FCRM), Brazzaville, Republic of Congo; 17 Institute for Tropical Medicine, University of Tübingen, Tübingen, Germany; 18 German Centre for Infection Research (DZIF), Partner Site Tübingen, Tübingen, Germany

**Keywords:** Malaria eradication, Compliance, Effectiveness, Existing antimalarial medicines, Malaria, Sulfadoxine–pyrimethamine, Artesunate–pyronaridine, Single-dose cure

## Abstract

Malaria remains a preventable and treatable disease; however, recent efforts to reduce mortality have plateaued. Although artemisinin-based combination therapy demonstrates high efficacy in controlled clinical settings, its real-world effectiveness is often compromised by suboptimal patient adherence. Specifically, the artemether–lumefantrine regimen, administered twice daily over 3 days, has been associated with reduced compliance due to its complexity. Simplified therapeutic regimens that enhance adherence could, therefore, play a critical role in reinvigorating progress toward malaria elimination. Over the past decade, substantial progress has been made in the discovery and development of new chemical entities for malaria treatment, although the most advanced candidate still requires a 3-day dosing regimen. Treatment shortening most likely requires multiple drug combinations. Multi-drug regimens, such as artemether–lumefantrine–amodiaquine appear to be well tolerated, but these are under development to address emerging resistance to lumefantrine and will be unlikely to improve compliance. Sulfadoxine–pyrimethamine was originally developed as a single-dose curative treatment for malaria, and although use was curtailed early due to rapid selection for resistance, it continues to be deployed as a single therapy or in combination with other medicines, in treatment and in prevention. Combining with artemisinin-based combinations would be an option for potential treatment shortening. Of the registered antimalarial treatments, only a few of the artemisinin-based combinations are suitable. Mefloquine is excluded for tolerability concerns, amodiaquine because of its use in seasonal malaria chemoprevention, and lumefantrine and piperaquine due to concerns of emerging resistance. Pyronaridine–artesunate emerges as a promising candidate for association with sulfadoxine–pyrimethamine. A four-drug, single-dose antimalarial regimen would transform compliance, and play a major role in disease elimination. However, to ensure its success it will be important to assess the safety and tolerability of the novel association and understand its efficacy in regions with evolving resistance to sulfadoxine–pyrimethamine. Clinical studies need to assess the risk for selection of strains with novel resistance mechanisms against artesunate or pyronaridine. Importantly, a comprehensive clinical evaluation will generate valuable real-world insights into community acceptance and operational feasibility. This information will be an important foundation for future design of single dose malaria therapies involving new chemical entities.

## Background

The 2023 burden of malaria was reported globally to an estimated 263 million cases, and an estimated 597,000 deaths [1]. These estimates suggest an overall increase of 11 million cases from the previous year. Africa continues to carry the heaviest burden of morbidity and mortality, accounting for about 95% of malaria cases and estimated malaria deaths. *Plasmodium falciparum* is the major parasitic species affecting humans in Africa. The most effective way to manage an uncomplicated case or to avoid progression to severe disease and death is through early diagnosis and prompt and efficacious treatment.

Artemisinin-based combination therapy (ACT), given for 3 days, show efficacy often far above the World Health Organization (WHO) recommended minimum threshold of 95% adequate clinical and parasitological response (ACPR) at day 28 during clinical studies. However, artemisinin-based combinations show lower effectiveness in real-world practice [2, 3], because of many system-specific issues. These include suboptimal intervention access, diagnostic shortcomings, incomplete provider compliance, but most importantly client adherence to the course of therapy. Many studies have shown that compliance rates with the 3-day ACT regimens are highly variable, but as low as 40% [4, 5].

With the development of antimalarial resistance to both artemisinins and many of the partner drugs, there is an urgent need for new therapies with new modes of action [6–8]. The first new approach was the development of synthetic peroxides. Arterolane (OZ277/Rbx11160) was successfully developed as a 3-day medicine, Synriam, in combination with piperaquine and has been widely used in India. Based on this success, the next generation artefenomel (OZ439) was developed as a potential single dose cure in combination with ferroquine. This only achieved 91% efficacy in a phase 2 study, and had significant galenic or formulation issues leading to poor tolerability in small infants [9, 10]. After this, the current most advanced new molecule is a formulation of ganaplacide (KAF156) and lumefantrine, which showed 92–94% efficacy in phase 2 as a single dose [11, 12]. Given the 100% cure rates with 3 days of dosing, this combination has now been tested in phase 3 as a 3-day dosing [13]. However, its potential for treatment shortening by adding a third drug is in early clinical exploration [14]. The other single-dose combinations that are still in early phases include cabamaquine (M5717) + pyronaridine [15], and ZY19489 + ferroquine [16], both of which are exploring both single and multiple dose options. In summary, there are many new combinations which are currently designed to overcome artesunate resistance, and some show potential for treatment shortening, but registration of any new shorter course treatments with new chemical entities will still need several more years.

## Triple and multidrug combinations as a platform for targeting treatment shortening

There has been a resurgence of interest in triple combinations in recent years: combining artesunate with two long-acting partner drugs, to provide a multiple-stage antimalarial activities (i.e. asexual blood stage, liver stage or sexual stage) and to maintain activity in the presence of partner drug resistance, and thus extend the clinical utility of artemisinin combinations [17–19, 24–26]. Several clinical trials on multidrug antimalarial combinations (MDACT) are underway or have been completed, and give indications for how this platform can be used for treatment shortening [24, 25]. For example, data from studies of artemether–lumefantrine–amodiaquine confirm that an effective combination can be developed that has high efficacy, and good safety and tolerability [28]. A combination of artemether–lumefantrine with atovaquone–proguanil is also being tested clinically [26]. However, not all combinations survive early clinical studies as was shown two decades ago by the failure of chlorproguanil–dapsone–artesunate [27], or more recently, attempts to combine atovaquone–proguanil with amodiaquine were stopped because of reports that atovaquone–proguanil can exacerbate a rare adverse event linked to amodiaquine [29]. This underlines that multidrug combinations are perfectly feasible, and can be well tolerated, but clinical data on combination safety and tolerability is essential.

## Exploring single-dose therapy using existing medicines

It is timely to consider whether combinations of existing drugs could shorten malaria treatment and improve compliance and effectiveness. The goal would be a regimen effective against both current and emerging resistant strains [17–19]. Combining four agents with distinct resistance mechanisms may help prevent the development of resistance—an approach long used in tuberculosis therapy empirically [20, 21]. Using approved drugs for such an approach also supports affordability and providing new medicines in the same price range as ACT for procurement agencies, such as the Global Fund [22]. However, single-dose cures pose significant pharmacological challenges [2, 23]. The dose for each ‘long acting’ drug must sustain curative plasma concentrations long enough to clear parasites, achieving curative drug levels for 6–8 days. There is additional complexity in the variability of bioavailability, and limited semi-immunity especially in children [2]. The additional drug loading in using four or more drugs as a single dose may increase adverse reactions like vomiting due to drug loading, risking subtherapeutic exposure.

## Chosing sulfadoxine–pyrimethamine as the basis for a treatment shortening multi-drug combination

Sulfadoxine–pyrimethamine (SP) has two active ingredients which target the enzymes, dihydropteroate synthase and dihydrofolate reductase in the folic acid synthesis pathway. It was the first antimalarial combination to cure uncomplicated *P. falciparum* malaria with a single dose, offering major operational advantages over multi-day regimens like chloroquine or quinine [30]. It was first was introduced in Thailand in 1967 (Fansidar®), but resistance appeared that same year and resistance spread quickly throughout South-East Asia. Resistance remained much lower in Africa: it was first launched in Malawi in 1993, but falling efficacy [31, 32] led to its replacement by ACT in the first decade of the millennium. It remains a WHO- recommended standard of care for treatment in many countries.

The correlation between mutations in the target genes dihydrofolate synthase and dihydropteroate synthase genes and clinical activity is not simple, since SP retains significant clinical activity even in the case of multidrug resistance [33, 34]. Factors such as immunity, drug metabolism, and infection multiplicity clearly influence outcomes. In chemoprevention studies, SP combinations remain active even in the presence of 90% of the quintuple mutation *dhps* [35]. It also shows high efficacy in chemoprevention, with no impact on efficacy in the presence of the quintuple mutations [35]. The widespread use of SP in chemoprevention is likely to mean that the resistance level is at best stable though, and will remain fixed in the parasite population in Africa [36], in contrast to the re-emergence of chloroquine-susceptible malaria after the removal of drug pressure [37]. Nonetheless, SP shows remarkable tenacity as a drug combination, and some of this tenacity is likely linked to the matched pharmacokinetics of the two drugs and their synergistic action [38].

SP has a good safety record: beyond its widespread use in treatment as SP + artesunate; it is used by over 20 million women to protect against infection in pregnancy (IPTp), over 50 million children under 5 years old in Seasonal Malaria Chemoprevention combination, where it shows a median efficacy of 75% [39]. New SP combinations for treatment cannot currently be introduced in areas where SP is used for prophylaxis. However, as vaccines, monoclonal antibodies, and long-acting injectables become more widely adopted, this limitation will diminish [33]. In summary, combining SP with other registered antimalarials could yield a well-tolerated, safe, effective, and deployable single-dose cure—bridging the gap until next-generation therapies arrive.

## Selection of a partner drug for sulfadoxine–pyrimethamine in multidrug combinations for the single dose cure and prevention of malaria?

There is limited scope for choosing a partner for SP. The only two other classes of drugs approved for treatment are atovaquone–proguanil and the artemisinin-based combinations. Use of atovaquone–proguanil as a partner drug is limited by the current price (linked to the cost of manufacture, and currently small market), and the relative lack of availability of safety data in African subjects or patients given the commercial focus in prophylaxis for travellers. Artemisinin-based combinations are more attractive as potential partners: artesunate itself has been widely used as a loose combination with SP, confirming the tolerability and safety of the combination, and historically showing high efficacy. The combination is relatively robust clinically, despite the presence of multiple *dhfr* and *dhps* mutations. Its showed 100% efficacy in India (as measured by PCR-corrected cure rates at day 28), despite the presence of 27% triple mutants [40], and high efficacy continues to be reported [41]. Even against a background of 56% quintuple mutations in Somalia, it still showed an efficacy of 87.7% [42]. These data underline the need for characterization of the *dhfr/dhps* resistant mutants in any clinical study, but confirm that SP still retains significant clinical efficacy against multi-resistant strains. It will also be important to characterize the impact of *kelch13* mutations, which will clearly impact the speed of resolution of infection and symptoms, although for uncomplicated malaria the link to any change in clinical efficacy is less clear.

Beyond artesunate + SP, there are five other combinations which are recommended by the WHO, based on the partner molecules lumefantrine, amodiaquine, piperaquine, mefloquine and pyronaridine. The combination with an artemisinin-based combination rather than the single aminoquinoline drug is a practical question: of the five drugs, only mefloquine is available as a monotherapy. Each partner drug merits consideration as the potential partner for a four-drug combination with SP and artemisinins. The strengths and weaknesses of the various artemisinin-based combinations are summarized below.

Artemether–lumefantrine (AL): has the disadvantage that in its current formulation requires to be dosed twice daily with food. Although a single daily dose formulation of lumefantrine has been developed by Novartis for ganaplacide–lumefantrine, no-one has so far developed the equivalent once per day dosing for artemether–lumefantrine [11, 12]. The half-life of lumefantrine and its metabolite is approximately 6 days, which is much shorter than the other ACT partner drugs (and their active metabolites). Therefore. it has a lower plasma concentration at days 28 and 42 leading to lower post-treatment prophylaxis than other ACT combination partners [43]. Lumefantrine resistance has been reported both in Uganda and in parasites cultured from a traveller recently returning from Uganda [44, 45]. On the positive side artemether–lumefantrine is the first ACT to be given positive guidance by the WHO for use in first trimester pregnancy: this is an important factor, given over 40 million pregnancies annually in Africa, and the fact that many cannot determine or communicate their pregnancy status.

Artesunate-amodiaquine (ASAQ): amodiaquine was originally linked to hepatotoxicity and agranulocytosis, leading to a black box warning by the US FDA. Widespread use as artesunate-amodiaquine has not confirmed this risk in African patients, and the main adverse events for the fixed dose combination ASAQ are QTc prolongation and a rare extrapyramidal syndrome. Concerns about amodiaquine resistance exist in West Africa, although these seem to have stabilized or even reversed with the delivery of fixed dose artesunate-amodiaquine since 2008.

DHA–piperaquine (DP): has been widely deployed, and piperaquine resistant parasites have been linked to clinical failure in South East Asia, and potentially in Africa [46, 47]. It has a considerable QTc effect, similar to chloroquine. It is given without food, but has a threefold food effect, meaning that if patients inadvertently take food, the plasma levels are threefold higher, leading to a QTc prolongation, which has raised some cardiac safety concerns for its use in mass drug administration [48]. The formulation of DHA–piperaquine requires that the two drugs be kept physically separate, and this has led to a significant cost differential between DP and earlier artemisinin-based combinations, such as AL and ASAQ.

Mefloquine–artesunate (ASMQ): showed poor tolerability when administered at high doses [49], and is significantly more expensive than other partner compounds. Resistance to mefloquine was reported in South East Asia leading to a switch to DHA–piperaquine. Mefloquine has been associated with an increased risk of mother to child transmission of HIV [50], and has a black box warning from the US FDA related to neuropsychiatric events. The relatively limited use means that ASMQ is typically the most expensive fixed dose ACT.

Artesunate–pyronaridine (AP): is the newest of the artemisinin-based combinations, given a positive scientific opinion by the European Medicines Agency in 2012 as a 3-day treatment for uncomplicated malaria and incorporated into the WHO treatment guidelines in 2023. Pyronaridine has the advantage of the absence of a major effect of food intake on bioavailability, and a long post-treatment protection effect. No resistance mutations have been identified in clinical studies [51], and despite extensive incubations, no resistance mutations have been reported from in vitro studies [52]. Pyronaridine has no significant effect on QTc prolongation (predicted to be less than 10 ms) [53]. It was originally associated with enhanced liver enzyme levels in repeat dose phase 1 volunteer studies, but more extensive studies in patients showed artesunate-pyronaridine and artemether–lumefantrine have similar hepatic safety when used repeatedly in participants with uncomplicated malaria [54]. That safety profile was further confirmed in a large real-world cohort even monitoring trial of more than 8500 malaria episodes [55]. Pyronaridine appears to be well tolerated in pregnancy. No teratogenic effects were observed in animals preclinically, and no increase in adverse pregnancy outcomes of teratogenic effects in 52 pregnancy outcomes from first trimester exposure [56], and with more data becoming available from ongoing interventional studies [57], and 839 pregnancies in second and third trimester (PYRAPREG consortium, http://www.pyrapreg.org) [58] and, therefore, is recommended by the European Medicines Agency in cases where artemether–lumefantrine or quinine–clindamycin are not available (EMA SmPC, Pyramax). A single dose of AP has been shown to clear infection in subjects with asymptomatic *P. falciparum* infection [59], with the caveat that the mean age was 16.6 years, suggesting they had acquired a certain immunity to malaria, and the geometric mean parasite density was low (550/µl). From a resistance perspective, then no stable pyronaridine resistance mutants have been selected and characterized in the laboratory, and pyronaridine is fully active against clinical isolates showing resistance to the other ACT partners.

Based on these data, the best candidate for a partner to SP for a single-dose cure with a multidrug combination therapy would be AP, based on its long half-life, good safety and tolerability record especially the emerging data in early pregnancy, lack of a food effect, and lack of detected resistance. This concept is currently evaluated in a proof of concept pilot study in Gabon assessing the efficacy, safety, and tolerability of the combination in children and adults with uncomplicated malaria (1-D-CURE trial, PACTR202405571736678).

## Cost of medicine and local manufacturing

Sulfadoxine-pyrimethamine (SPAP) as a potential single dose cure is attractive from a cost perspective. Based on current pricing through the Global Fund Process, a single adult dose of SPAP could be priced around $0.95 for an adult [60], which is only slightly higher than a 3 day adult course of AL at $0.60. In addition, the cost of new medicines drops substantially post-launch: artemether–lumefantrine for example is priced fourfold lower than public health launch cost of $2.40 in 2001. If similar savings could be made for artesunate–pyronaridine, a price of $0.45 per adult could be achievable, making it extremely interesting compared to 3 days of ACT.

Beyond the simple question of price, in the current funding climate there is also the question of local manufacturing. National Malaria Control Programmes will become increasingly dependent on the use of national and local funds to procure medicines, and locally manufactured medicines will become increasingly important. SP is already manufactured in Africa to the international manufacturing ICH GMP standards, and approved for WHO prequalification. Artesunate–pyronaridine is currently only being manufactured by Shin Poong in South Korea, and there are now projects to support its manufacturing in Africa.

## Ethical implications of implementing a single encounter cure for malaria with existing antimalarial drugs

The expected benefits of single dose therapy include improved compliance, with higher effectiveness, but also decreased costs, both important for malaria elimination. A single dose cure could dramatically improve access to medicines in remote areas, and a key question will be ensuring equitable distribution, where local manufacturing will be key. Whilst the safety and tolerability of the two two-drug combinations are understood, the potential for rare, but unexpected new adverse events must be managed by a stepwise increase in the number of cases treated to gain robust evidence of safety and tolerability. Probably the most important ethical question will be to manage the development to ensure that the maximum knowledge about the potential for new resistance selection is obtained, in particular resistance against pyronaridine. Establishing the efficacy and effectiveness of SPAP especially in areas where the more severe quintuple mutation SP resistance is not particularly widespread will be an important first step. SPAP will help to refine the target product profile for a single dose cure, and serve as a benchmark for future treatment shortening regimens, to focus on providing evidence for better efficacy, tolerability and cost-effectiveness.

## Conclusion

Despite good progress in the early part of the century, the malaria burden has recently plateaued or increased. One of the reasons for this is that although the clinical efficacy of antimalarials remains high, real-life effectiveness is clearly lower, driven by incomplete compliance to a 3-day treatment. Although there are many new drugs in development, in the short term there is a critical opportunity to optimize the use of existing drugs to simplify malaria treatment and improved compliance. The existing drugs have the advantage that their safety profiles are well understood, including data on key populations such as small infants and pregnant women. The existing drugs tend to be more cost effective than new drugs, and many are being manufactured in Africa. More effective case management of malaria cannot be postponed until the next generation of new chemical entities will become available; there is a need now, as shown by the stalling progress of malaria elimination. Sulfadoxine–pyrimethamine forms a good basis for a new multi-drug regimen, given that it targets two mechanisms of action which are different from the artemisinin-based combinations. It will be important to investigate to what extent SP in multi-drug combinations is compromised by new resistance mutations, and to determine whether these are significant enough to put the clinical target of 95% ACPR at day 28 beyond reach. Other gaps that need to be addressed in the development of SPAP, include the absence of modelled pharmacokinetic/pharmacodynamic parameters for single-dose administration across age groups; and to obtain more clinical safety data on the SPAP combination. These are questions that can only be answered by clinical data, and this is where the hypothesis needs to be tested. If the control of malaria is to get back on track, there is no time to lose.

## Data Availability

No datasets were generated or analysed during the current study.

## Abbreviations

ACPR: Adequate clinical and parasitological response
ACT: Artemisinin-based combination therapy
AL: Artemether–lumefantrine
AP: Artesunate–pyronaridine
ASAQ: Artesunate–amodiquine
DP: Dihydroartemisinin–piperaquine
IPTp: Intermittent preventive treatment in pregnancy
MDACT: Multidrug antimalarial combination therapy
SP: Sulfadoxine–pyrimethamine
SPAP: Sulfadoxine–pyrimethamine plus artesunate–pyronaridine
WHO: World Health Organization

## References

[1] WHO. World malaria report 2024. Geneva : World Health Organization ; 2024. https://www.who.int/teams/global-malaria-programme/reports/world-malaria-report-2024. Accessed 17 May 2025.

[2] Mbassi DE, Pfaffendorf C, Mombo-Ngoma G, Kreuels B, Ramharter M. Real-life effectiveness of anti-malarial treatment regimens: what are we aiming for? Malar J. 2023;22:189. 37340324 10.1186/s12936-023-04606-2 PMC10280843

[3] The malERA Consultative Group on Drugs. A research agenda for malaria eradication: drugs. PLoS Med. 2011;8:e1000402. 21311580 10.1371/journal.pmed.1000402 PMC3026688

[4] Banek K, Lalani M, Staedke SG, Chandramohan D. Adherence to artemisinin-based combination therapy for the treatment of malaria: a systematic review of the evidence. Malar J. 2014;13:7. 24386988 10.1186/1475-2875-13-7 PMC3893456

[5] Bruxvoort K, Festo C, Cairns M, Kalolella A, Mayaya F, Kachur SP, et al. Measuring patient adherence to malaria treatment: a comparison of results from self-report and a customised electronic monitoring device. PLoS ONE. 2015;10:e0134275. 26214848 10.1371/journal.pone.0134275 PMC4516331

[6] Martin RE, Shafik SH, Richards SN. Mechanisms of resistance to the partner drugs of artemisinin in the malaria parasite. Curr Opin Pharmacol. 2018;42:71–80. 30142480 10.1016/j.coph.2018.07.010

[7] Okwu DG, Zoleko Manego R, Duparc S, Kremsner PG, Ramharter M, Mombo-Ngoma G. The non-artemisinin antimalarial drugs under development: a review. Clin Microbiol Infect. 2025;31:941–7. 40120754 10.1016/j.cmi.2025.03.009

[8] Alonso PL, Brown G, Arevalo-Herrera M, Binka F, Chitnis C, Collins F, et al. A research agenda to underpin malaria eradication. PLoS Med. 2011;8:e1000406. 21311579 10.1371/journal.pmed.1000406 PMC3026687

[9] Adoke Y, Zoleko-Manego R, Ouoba S, Tiono AB, Kaguthi G, Bonzela JE, et al. A randomized, double-blind, phase 2b study to investigate the efficacy, safety, tolerability and pharmacokinetics of a single-dose regimen of ferroquine with artefenomel in adults and children with uncomplicated *Plasmodium falciparum* malaria. Malar J. 2021;20:222. 34011358 10.1186/s12936-021-03749-4 PMC8135182

[10] Gansane A, Lingani M, Yeka A, Nahum A, Bouyou-Akotet M, Mombo-Ngoma G, et al. Randomized, open-label, phase 2a study to evaluate the contribution of artefenomel to the clinical and parasiticidal activity of artefenomel plus ferroquine in African patients with uncomplicated *Plasmodium falciparum* malaria. Malar J. 2023;22:2. 36597076 10.1186/s12936-022-04420-2 PMC9809015

[11] Ogutu B, Yeka A, Kusemererwa S, Thompson R, Tinto H, Toure AO, et al. Ganaplacide (KAF156) plus lumefantrine solid dispersion formulation combination for uncomplicated *Plasmodium falciparum* malaria: an open-label, multicentre, parallel-group, randomised, controlled, phase 2 trial. Lancet Infect Dis. 2023;23:1051–61. 37327809 10.1016/S1473-3099(23)00209-8

[12] Sangana R, Ogutu B, Yeka A, Kusemererwa S, Tinto H, Toure AO, et al. Pharmacokinetics of ganaplacide and lumefantrine in adults, adolescents, and children with *Plasmodium falciparum* malaria treated with ganaplacide plus lumefantrine solid dispersion formulation: analysis of data from a multinational phase 2 study. J Clin Pharmacol. 2025;65:179–89. 39344281 10.1002/jcph.6138 PMC11771541

[13] Novartis Pharmaceuticals. A phase 2 interventional, multicenter, randomized open label study to determine the effective and tolerable dose of KAF156 and lumefantrine solid dispersion formulation in combination, given once daily for 1, 2 and 3-days to adults and children with uncomplicated *Plasmodium falciparum* malaria. clinicaltrials.gov; 2021 Dec. Report No.: NCT03167242. https://clinicaltrials.gov/study/NCT03167242

[14] Novartis Pharmaceuticals. A randomized, open-label, multicenter study to compare efficacy, safety and tolerability of KLU156 with Coartem® in the treatment of uncomplicated *Plasmodium falciparum* malaria in adults and children ≥ 5 kg body weight followed by an extension phase with repeated KLU156 treatment. clinicaltrials.gov; 2025 May. Report No.: NCT05842954. https://clinicaltrials.gov/study/NCT05842954

[15] Merck Healthcare KGaA, Darmstadt, Germany, an affiliate of Merck KGaA, Darmstadt, Germany. Phase IIa proof of concept, multicenter, randomized, open-label study to evaluate the safety, efficacy, and pharmacokinetics of the combination M5717 plus pyronaridine administered once daily for 1 or 2 days to adults and adolescents with acute uncomplicated *Plasmodium falciparum* malaria (CAPTURE 1). clinicaltrials.gov; 2025 Jan. Report No.: NCT05689047. https://clinicaltrials.gov/study/NCT05689047

[16] Zydus Lifesciences Limited. Phase Ib, single-center, randomized, study to determine safety, tolerability, and pharmacokinetics of different orally administered regimens of the combination ZY19489-ferroquine in adult asymptomatic *Plasmodium falciparum* carriers. clinicaltrials.gov; 2024 Sep. Report No.: NCT05911828. https://clinicaltrials.gov/study/NCT05911828

[17] Bassat Q, Maïga-Ascofaré O, May J, Clain J, Mombo-Ngoma G, Groger M, et al. Challenges in the clinical development pathway for triple and multiple drug combinations in the treatment of uncomplicated falciparum malaria. Malar J. 2022;21:61. 35193586 10.1186/s12936-022-04079-9 PMC8864855

[18] van der Pluijm RW, Amaratunga C, Dhorda M, Dondorp AM. Triple artemisinin-based combination therapies for malaria - a new paradigm? Trends Parasitol. 2021;37:15–24. 33060063 10.1016/j.pt.2020.09.011

[19] Nguyen TD, Gao B, Amaratunga C, Dhorda M, Tran TNA, White NJ, et al. Preventing antimalarial drug resistance with triple artemisinin-based combination therapies. Nat Commun. 2023;14:4568. 37516752 10.1038/s41467-023-39914-3 PMC10387089

[20] Larkins-Ford J, Aldridge BB. Advances in the design of combination therapies for the treatment of tuberculosis. Expert Opin Drug Discov. 2023;18:83–97. 36538813 10.1080/17460441.2023.2157811 PMC9892364

[21] Woods RJ, Read AF. Combination antimicrobial therapy to manage resistance. Evol Med Public Health. 2023;11:185–6. 37360836 10.1093/emph/eoad005 PMC10287501

[22] Institute of Medicine (US) committee on the economics of antimalarial drugs, Arrow KJ, Panosian C, Gelband H. Executive Summary. Saving lives, buying time: economics of malaria drugs in an age of resistance. National Academies Press (US); 2004. https://www.ncbi.nlm.nih.gov/books/NBK215623/. Accessed 9 Aug 2025. 25009879

[23] White NJ, Nosten FH. SERCAP: is the perfect the enemy of the good? Malar J. 2021;20:281. 34167536 10.1186/s12936-021-03821-z PMC8223304

[24] van der Pluijm RW, Tripura R, Hoglund RM, Pyae Phyo A, Lek D, ul Islam A, et al. Triple artemisinin-based combination therapies versus artemisinin-based combination therapies for uncomplicated *Plasmodium falciparum* malaria: a multicentre, open-label, randomised clinical trial. Lancet. 2020;395:1345–60. 32171078 10.1016/S0140-6736(20)30552-3 PMC8204272

[25] Peto TJ, Tripura R, Callery JJ, Lek D, Nghia HDT, Nguon C, et al. Triple therapy with artemether-lumefantrine plus amodiaquine versus artemether-lumefantrine alone for artemisinin-resistant, uncomplicated falciparum malaria: an open-label, randomised, multicentre trial. Lancet Infect Dis. 2022;22:867–78. 35276064 10.1016/S1473-3099(21)00692-7 PMC9132777

[26] Clinical Trial. ASAAP. https://asaap-malaria.org/research-platform/clinical-trial/. Accessed 17 May 2025.

[27] Tiono AB, Dicko A, Ndububa DA, Agbenyega T, Pitmang S, Awobusuyi J, et al. Chlorproguanil-dapsone-artesunate versus chlorproguanil-dapsone: a randomized, double-blind, phase III trial in African children, adolescents, and adults with uncomplicated *Plasmodium falciparum* malaria. Am J Trop Med Hyg. 2009;81:969–78. 19996424 10.4269/ajtmh.2009.09-0351

[28] Mahamar A, Vanheer LN, Smit MJ, Sanogo K, Sinaba Y, Niambele SM, et al. Artemether–lumefantrine–amodiaquine or artesunate–amodiaquine combined with single low-dose primaquine to reduce *Plasmodium falciparum* malaria transmission in Ouélessébougou, Mali: a five-arm, phase 2, single-blind, randomised controlled trial. Lancet Microbe. 2025;6:100966. 39701119 10.1016/j.lanmic.2024.100966 PMC11798902

[29] Chalon S, Chughlay MF, Abla N, Marie Tchouatieu A, Haouala A, Hutter B, et al. Unanticipated CNS safety signal in a placebo-controlled, randomized trial of co-administered atovaquone-proguanil and amodiaquine. Clin Pharmacol Ther. 2022;111:867–77. 34453327 10.1002/cpt.2404 PMC9291514

[30] Sulfadoxine/Pyrimethamine—an overview. ScienceDirect Topics. https://www.sciencedirect.com/topics/neuroscience/sulfadoxine-pyrimethamine. Accessed 17 May 2025.

[31] Roper C, Pearce R, Nair S, Sharp B, Nosten F, Anderson T. Intercontinental spread of pyrimethamine-resistant malaria. Science. 2004;305:1124. 15326348 10.1126/science.1098876

[32] Pearce RJ, Pota H, Evehe M-SB, Bâ E-H, Mombo-Ngoma G, Malisa AL, et al. Multiple origins and regional dispersal of resistant dhps in African *Plasmodium falciparum* malaria. PLoS Med. 2009;6:e1000055. 19365539 10.1371/journal.pmed.1000055 PMC2661256

[33] El Gaaloul M, Tchouatieu AM, Kayentao K, Campo B, Buffet B, Ramachandruni H, et al. Chemoprevention of malaria with long-acting oral and injectable drugs: an updated target product profile. Malar J. 2024;23:315. 39425110 10.1186/s12936-024-05128-1 PMC11490162

[34] Mbugi EV, Mutayoba BM, Malisa AL, Balthazary ST, Nyambo TB, Mshinda H. Drug resistance to sulphadoxine-pyrimethamine in *Plasmodium falciparum* malaria in Mlimba, Tanzania. Malar J. 2006;5:94. 17076899 10.1186/1475-2875-5-94 PMC1636063

[35] van Eijk AM, Larsen DA, Kayentao K, Koshy G, Slaughter DEC, Roper C, et al. Effect of *Plasmodium falciparum* sulfadoxine-pyrimethamine resistance on the effectiveness of intermittent preventive therapy for malaria in pregnancy in Africa: a systematic review and meta-analysis. Lancet Infect Dis. 2019;19:546–56. 30922818 10.1016/S1473-3099(18)30732-1

[36] Artimovich E, Schneider K, Taylor TE, Kublin JG, Dzinjalamala FK, Escalante AA, et al. Persistence of sulfadoxine-pyrimethamine resistance despite reduction of drug pressure in Malawi. J Infect Dis. 2015;212:694–701. 25672905 10.1093/infdis/jiv078 PMC4539899

[37] Frosch AEP, Laufer MK, Mathanga DP, Takala-Harrison S, Skarbinski J, Claassen CW, et al. Return of widespread chloroquine-sensitive *Plasmodium falciparum* to Malawi. J Infect Dis. 2014;210:1110–4. 24723474 10.1093/infdis/jiu216 PMC6281358

[38] Divala TH, Cohee LM, Laufer MK. The remarkable tenacity of sulfadoxine-pyrimethamine. Lancet Infect Dis. 2019;19:460–1. 30922819 10.1016/S1473-3099(18)30796-5 PMC10321323

[39] Masserey T, Lee T, Kelly SL, Hastings IM, Penny MA. Seasonal malaria chemoprevention and the spread of *Plasmodium falciparum* quintuple-mutant parasites resistant to sulfadoxine–pyrimethamine: a modelling study. Lancet Microbe. 2024;5:100892. 38996497 10.1016/S2666-5247(24)00115-0

[40] Srivastava P, Ratha J, Shah NK, Mishra N, Anvikar AR, Sharma SK, et al. A clinical and molecular study of artesunate + sulphadoxine-pyrimethamine in three districts of central and eastern India. Malar J. 2013;12:247. 23866298 10.1186/1475-2875-12-247 PMC3726327

[41] Arya A, Kojom Foko LP, Chaudhry S, Sharma A, Singh V. Artemisinin-based combination therapy (ACT) and drug resistance molecular markers: a systematic review of clinical studies from two malaria endemic regions – India and sub-Saharan Africa. Int J Parasitol Drugs Drug Resist. 2021;15:43–56. 33556786 10.1016/j.ijpddr.2020.11.006 PMC7887327

[42] Warsame M, Hassan AH, Hassan AM, Arale AM, Jibril AM, Mohamud SA, et al. Efficacy of artesunate + sulphadoxine/pyrimethamine and artemether + lumefantrine and *dhfr* and *dhps* mutations in Somalia: evidence for updating the malaria treatment policy. Trop Med Int Health. 2017;22:415–22. 28151566 10.1111/tmi.12847

[43] Bretscher MT, Dahal P, Griffin J, Stepniewska K, Bassat Q, Baudin E, et al. The duration of chemoprophylaxis against malaria after treatment with artesunate-amodiaquine and artemether-lumefantrine and the effects of *pfmdr1* 86Y and *pfcrt* 76T: a meta-analysis of individual patient data. BMC Med. 2020;18:47. 32098634 10.1186/s12916-020-1494-3 PMC7043031

[44] van Schalkwyk DA, Pratt S, Nolder D, Stewart LB, Liddy H, Muwanguzi-Karugaba J, et al. Treatment failure in a UK malaria patient harboring genetically variant *Plasmodium falciparum* from Uganda with reduced in vitro susceptibility to artemisinin and lumefantrine. Clin Infect Dis. 2024;78:445–52. 38019958 10.1093/cid/ciad724 PMC10874266

[45] Tumwebaze PK, Conrad MD, Okitwi M, Orena S, Byaruhanga O, Katairo T, et al. Decreased susceptibility of *Plasmodium falciparum* to both dihydroartemisinin and lumefantrine in northern Uganda. Nat Commun. 2022;13:6353. 36289202 10.1038/s41467-022-33873-x PMC9605985

[46] Pernaute-Lau L, Recker M, Tékété M, de Sousa TN, Traore A, Fofana B, et al. Decreased dihydroartemisinin-piperaquine protection against recurrent malaria associated with *Plasmodium falciparum* plasmepsin 3 copy number variation in Africa. Nat Commun. 2025;16:2680. 40102390 10.1038/s41467-025-57726-5 PMC11920258

[47] Sagara I, Beavogui AH, Zongo I, Soulama I, Borghini-Fuhrer I, Fofana B, et al. Pyronaridine–artesunate or dihydroartemisinin–piperaquine versus current first-line therapies for repeated treatment of uncomplicated malaria: a randomised, multicentre, open-label, longitudinal, controlled, phase 3b/4 trial. Lancet. 2018;391:1378–90. 29606364 10.1016/S0140-6736(18)30291-5 PMC5889791

[48] Wattanakul T, Ogutu B, Kabanywanyi AM, Asante K-P, Oduro A, Adjei A, et al. Pooled multicenter analysis of cardiovascular safety and population pharmacokinetic properties of piperaquine in African patients with uncomplicated falciparum malaria. Antimicrob Agents Chemother. 2020;64:e01848–19. 32312783 10.1128/AAC.01848-19 PMC7318010

[49] González R, Mombo-Ngoma G, Ouédraogo S, Kakolwa MA, Abdulla S, Accrombessi M, et al. Intermittent preventive treatment of malaria in pregnancy with mefloquine in HIV-negative women: a multicentre randomized controlled trial. PLoS Med. 2014;11:e1001733. 25247709 10.1371/journal.pmed.1001733 PMC4172436

[50] González R, Desai M, Macete E, Ouma P, Kakolwa MA, Abdulla S, et al. Intermittent preventive treatment of malaria in pregnancy with mefloquine in HIV-infected women receiving cotrimoxazole prophylaxis: a multicenter randomized placebo-controlled trial. PLoS Med. 2014;11:e1001735. 25247995 10.1371/journal.pmed.1001735 PMC4172537

[51] Sauthier S, Lazrek Y, Volney B, Mathieu L, Florimond C, Carod J-F, et al. Absence of *Plasmodium falciparum* resistance to pyronaridine *in vitro* over 2009–2023 in French Guiana. Médecine et Maladies Infectieuses Formation. 2024;3:S22.

[52] Chu W-Y, Dorlo TPC. Pyronaridine: a review of its clinical pharmacology in the treatment of malaria. J Antimicrob Chemother. 2023;78:2406–18. 37638690 10.1093/jac/dkad260 PMC10545508

[53] Croft SL, Duparc S, Arbe-Barnes SJ, Craft JC, Shin C-S, Fleckenstein L, et al. Review of pyronaridine anti-malarial properties and product characteristics. Malar J. 2012;11:270. 22877082 10.1186/1475-2875-11-270 PMC3483207

[54] Compaoré YD, Zongo I, Somé AF, Barry N, Nikiéma F, Kaboré TN, et al. Hepatic safety of repeated treatment with pyronaridine-artesunate versus artemether–lumefantrine in patients with uncomplicated malaria: a secondary analysis of the WANECAM 1 data from Bobo-Dioulasso, Burkina Faso. Malar J. 2021;20:64. 33514368 10.1186/s12936-021-03593-6 PMC7847156

[55] Tona Lutete G, Mombo-Ngoma G, Assi S-B, Bigoga JD, Koukouikila-Koussounda F, Ntamabyaliro NY, et al. Pyronaridine-artesunate real-world safety, tolerability, and effectiveness in malaria patients in 5 African countries: a single-arm, open-label, cohort event monitoring study. PLoS Med. 2021;18:e1003669. 34129601 10.1371/journal.pmed.1003669 PMC8205155

[56] Liverpool School of Tropical Medicine. A pregnancy registry to assess the safety of antimalarial use in pregnancy. clinicaltrials.gov; 2021 Mar. Report No.: NCT04825782. https://clinicaltrials.gov/study/NCT04825782

[57] Efficacy and Safety of a newly registered Artemisinin-Based Combination (Pyronaridine-Artesunate -PYRAMAX®) for the treatment of uncomplicated Malaria in African pregnant women — ERA-LEARN. https://www.era-learn.eu/network-information/networks/edctp-ii/clinical-trials-and-operational-research-studies-to-optimise-the-use-of-products-for-poverty-related-diseases-in-mothers-newborns-children-and-or-adolescents/efficacy-and-safety-of-a-newly-registered-artemisinin-based-combination-pyronaridine-artesunate-pyramax-r-for-the-treatment-of-uncomplicated-malaria-in-african-pregnant-women. Accessed 11 Aug 2025.

[58] Tshiongo JK, Khote FL, Kabena M, Mavoko HM, Kalonji-Mukendi T, Luzolo L, et al. Intermittent screening using ultra-sensitive malaria rapid diagnostic test and treatment with pyronaridine-artesunate compared to standard preventive treatment with sulfadoxine-pyrimethamine for malaria prevention in pregnant women in Kinshasa, DRC. Malar J. 2025;24:58. 39985024 10.1186/s12936-025-05260-6 PMC11846385

[59] Dabira ED, Hachizovu S, Conteh B, Mendy A, Nyang H, Lawal B, et al. Efficacy, safety and tolerability of pyronaridine-artesunate in asymptomatic malaria-infected individuals: a randomized controlled trial. Clin Infect Dis. 2022;74:180–8. 33983371 10.1093/cid/ciab425 PMC8800175

[60] Workbook: Price & Quality Reporting Price Reference Report. https://insights.theglobalfund.org/t/Public/views/PriceQualityReportingPriceReferenceReport/PriceList?iframeSizedToWindow=true&%3Aembed=y&%3AshowAppBanner=false&%3Adisplay_count=no&%3AshowVizHome=no. Accessed 19 May 2025.

